# A Single-Dose Combination Study with the Experimental Antimalarials Artefenomel and DSM265 To Determine Safety and Antimalarial Activity against Blood-Stage Plasmodium falciparum in Healthy Volunteers

**DOI:** 10.1128/AAC.01371-19

**Published:** 2019-12-20

**Authors:** James S. McCarthy, Thomas Rückle, Suzanne L. Elliott, Emma Ballard, Katharine A. Collins, Louise Marquart, Paul Griffin, Stephan Chalon, Jörg J. Möhrle

**Affiliations:** a QIMR Berghofer Medical Research Institute, Brisbane, Queensland, Australia; b Medicines for Malaria Venture, Meyrin, Switzerland; c Q-Pharm, Pty., Ltd., Brisbane, Queensland, Australia; d Department of Medicine and Infectious Diseases, Mater Hospital and Mater Research, Raymond Terrace, South Brisbane, Queensland, Australia; e The University of Queensland, Brisbane, Queensland, Australia

**Keywords:** DSM265, *Plasmodium falciparum*, artefenomel, chemotherapy, clinical studies, combination therapy, controlled human malaria infection, malaria, volunteer infection study

## Abstract

Artefenomel and DSM265 are two new compounds that have been shown to be well tolerated and effective when administered as monotherapy malaria treatment. This study aimed to determine the safety, pharmacokinetics, and pharmacodynamics of artefenomel and DSM265 administered in combination to healthy subjects in a volunteer infection study using the Plasmodium falciparum-induced blood-stage malaria model. Thirteen subjects were inoculated with parasite-infected erythrocytes on day 0 and received a single oral dose of artefenomel and DSM265 on day 7. Cohort 1 (*n* = 8) received 200 mg artefenomel plus 100 mg DSM265, and cohort 2 (*n* = 5) received 200 mg artefenomel plus 50 mg DSM265. Blood samples were collected to measure parasitemia, gametocytemia, and artefenomel-DSM265 plasma concentrations. There were no treatment-related adverse events. The pharmacokinetic profiles of artefenomel and DSM265 were similar to those of the compounds when administered as monotherapy, suggesting no pharmacokinetic interactions. A reduction in parasitemia occurred in all subjects following treatment (log_10_ parasite reduction ratios over 48 h [PRR_48_] of 2.80 for cohort 1 and 2.71 for cohort 2; parasite clearance half-lives of 5.17 h for cohort 1 and 5.33 h for cohort 2). Recrudescence occurred in 5/8 subjects in cohort 1 between days 19 and 28 and in 5/5 subjects in cohort 2 between days 15 and 22. Low-level gametocytemia (1 to 330 female gametocytes/ml) was detected in all subjects from day 14. The results of this single-dosing combination study support the further clinical development of the use of artefenomel and DSM265 in combination as a treatment for falciparum malaria. (This study has been registered at ClinicalTrials.gov under identifier NCT02389348.)

## Abstract (precis)

Artefenomel and DSM265 are two new compounds that have been shown to be well tolerated and effective when administered as monotherapy malaria treatment. This study aimed to determine the safety, pharmacokinetics, and pharmacodynamics of artefenomel and DSM265 administered in combination to healthy subjects in a volunteer infection study using the Plasmodium falciparum-induced blood-stage malaria model.

## INTRODUCTION

The World Health Organization has declared malaria eradication a global development priority. The emergence of drug-resistant parasites is a significant obstacle to malaria eradication and necessitates the clinical development of new antimalarial candidates. The use of combinations of drugs with unrelated modes of action reduces the risk of selecting for resistant mutants; thus, combination therapies are the focus of antimalarial drug development. Additional desirable properties of new antimalarial therapies include that they cure patients after a single administration (due to compliance challenges with multidose treatments in the field) and also that they block transmission of parasites to vector mosquitoes (1).

The early evaluation of potential antimalarial drugs in healthy participants following inoculation with blood-stage Plasmodium falciparum has a number of benefits. Volunteer infection studies (VIS) using the induced blood-stage malaria (IBSM) model allow for early demonstration of pharmacological activity (or lack of) in human subjects enabling go/no-go decision for new drug candidates. Determination of a compound’s pharmacokinetic/pharmacodynamic (PK/PD) relationship using VIS informs choice of dose in future studies such as phase 2 field trials and early assessment of the compound’s tolerability in a controlled disease-like setting. VIS have been used successfully to evaluate the safety and antimalarial activity of new antimalarial drug candidates in monotherapy (2–7). The model also has the potential to be used to investigate the PK/PD interactions between two or more compounds when coadministered, as well as to determine the tolerability and antimalarial activity of the combination and to inform dose selection for future trials.

Artefenomel, previously known as OZ439, is a synthetic ozonide that has been shown in preclinical studies (8) and in phase 1 and 2 clinical studies (2, 9, 10) to be a promising new peroxide anti-malarial agent. Ozonides are thought to act in a similar manner to the artemisinins by reacting with iron within the parasite food vacuole to produce free radicals, leading to alkylation of key parasitic proteins (11). DSM265 is a novel triazolopyrimidine-based inhibitor of the pyrimidine biosynthetic enzyme dihydroorotate dehydrogenase (DHODH). DHODH is an enzyme that is essential to the malaria parasite, as pyrimidine salvage pathways are absent in this organism and it thus relies on *de novo* synthesis to supply pyrimidine requirements (12). Preclinical studies indicated that DSM265 is highly selective toward *Plasmodium* DHODH and is active against both blood and liver stages of P. falciparum (13). Clinical studies have also been performed which have demonstrated the good tolerability profile of DSM265 in humans and its activity in clearing P. falciparum parasitemia (4, 14–16).

In recently completed VIS, both compounds demonstrated activity against P. falciparum blood-stage malaria parasites when administered as single doses in monotherapy. Artefenomel was found to be fast acting, with a rapid reduction in parasitemia observed following administration of a single dose of 200 or 500 mg (parasite clearance half-lives of 6.5 and 3.6 h, respectively), although recrudescence occurred in all subjects dosed with 200 mg and in 50% of subjects dosed with 500 mg (2). DSM265 was found to be slower in its effect on the clearance of parasites from the blood of the infected volunteers, with parasite clearance half-lives of 9.4 h following administration of a single dose of 150 mg (4) (recrudescence occurred in all subjects) and 5.2 h following administration of a single dose of 400 mg (no recrudescence occurred) (15). Furthermore, the PK profile of DSM265 indicates that parasiticidal concentrations in the blood are likely to be maintained for an extended period of time after administration of a single dose (4). These properties and the different modes of action of these compounds suggest that they may represent a good combination antimalarial treatment. The aim of the current study was to evaluate the safety, tolerability, PK, and PD of a single dose of artefenomel and DSM265 administered in combination to healthy participants using a P. falciparum VIS. Furthermore, the tendency of artefenomel-DSM265 combination treatment to induce gametocytemia in the blood of subjects was evaluated.

## RESULTS

### Subject disposition.

A total of 13 healthy subjects were enrolled in the study and inoculated with P. falciparum blood-stage parasites on day 0 (8 subjects in cohort 1 and 5 subjects in cohort 2). Only 5 subjects were enrolled in cohort 2 instead of the planned 8 subjects because of recruitment limitations. Furthermore, the sponsor decided after the second cohort that a third cohort was not necessary to meet the study objectives. The demographic characteristics of subjects are presented in Table 1. The majority of subjects were male (61.5%) and Caucasian (92.3%); the mean age of subjects was approximately 26 years. All 13 subjects were treated with a single dose of artefenomel-DSM265 on day 7 with a dose of 200 mg/100 mg for cohort 1 or 200 mg/50 mg for cohort 2. All 13 subjects received rescue treatment with artemether-lumefantrine on day 28 or before in the case of recrudescence of parasitemia.

**TABLE 1:** Demographic profile of subjects

Parameter	No. (%)^*a*^		
	Cohort 1 (*n* = 8)	Cohort 2 (*n* = 5)	Total (*n* = 13)
Age, yr			
Mean ± SD	27.8 ± 12.4	23.4 ± 2.3	26.1 ± 9.8
Range	19–55	21–27	19–55
Sex			
Male	5 (62.5)	3 (60.0)	8 (61.5)
Female	3 (37.5)	2 (40.0)	5 (38.5)
Race			
Caucasian	7 (87.5)	5 (100.0)	12 (92.3)
Latino	1 (12.5)	0 (0.0)	1 (7.7)
BMI,^*b*^ kg/m^2^			
Mean ± SD	23.5 ± 2.9	23.8 ± 2.4	23.6 ± 2.6
Range	19.9–28.1	21.3–27.4	19.9–28.1
Ht, cm			
Mean ± SD	173.0 ± 8.7	176.2 ± 3.1	174.2 ± 7.0
Range	163–187	171–179	163–187
Wt, kg			
Mean ± SD	70.9 ± 13.5	73.7 ± 7.5	72.0 ± 11.3
Range	55.9–87.1	67.5–85.8	55.9–87.1

a Dose: cohort 1, 200 mg artefenomel plus 100 mg DSM265; cohort 2, 200 mg artefenomel plus 50 mg DSM265.

b BMI, body mass index.

### Safety.

There was a total of 107 adverse events (AEs) reported during the study; all 13 subjects reported at least one AE (see Table S1 in the supplemental material). A large proportion of AEs (68.2%) were considered to be related to malaria; none were considered to be related to treatment with artefenomel and DSM265. The most common AEs were headache (*n* = 10 subjects), fatigue (*n* = 9 subjects), and myalgia and malaise (*n* = 7 subjects each). One subject experienced two severe AEs; laboratory results revealed elevated aspartate transaminase (10.9 times the upper limit of normal [ULN]) and elevated creatine kinase (170.2 × ULN). Concurrent mild elevations in alanine transaminase (3.6 × ULN) and lactate dehydrogenase (4.8 × ULN) were also observed for this subject. These elevations were not associated with any symptoms and were not considered to be related to any of the study interventions (malaria challenge agent or investigational compounds), but were attributed to the fact that the subject commenced a weightlifting program during the study after previously being inactive. The effect of weightlifting on liver function tests has been documented (17). Mild elevations in both alanine transaminase and aspartate transaminase (maximum of 5.3 × ULN and 3.9 × ULN, respectively) were also observed in two other subjects and were considered to be related to malaria. There were no serious adverse events reported in the study, and no AEs resulted in study discontinuation.

### Pharmacokinetics.

**FIG 1:**
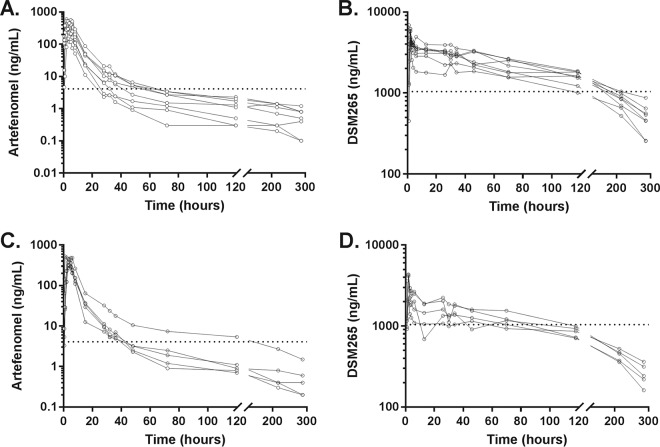
Individual subject plasma concentration-time profiles for artefenomel and DSM265. Subjects received a single dose of artefenomel-DSM265 on day 7. Cohort 1 received a dose of 200 mg artefenomel plus 100 mg DSM265, and cohort 2 received a dose of 200 mg artefenomel plus 50 mg DSM265. Lines indicate the artefenomel (A and C) and DSM256 (B and D) concentrations in the plasma over time for each subject in cohort 1 (A and B) and cohort 2 (C and D). The horizontal dotted line indicates the MIC calculated in previous IBSM studies (2, 4).

The mean peak artefenomel plasma concentration in cohort 1 was 372 ng/ml, which occurred 3 h postadministration (Fig. 1). The estimated total exposure (area under the concentration-time curve from 0 h to infinity [AUC_0–∞_]) to artefenomel was 3,083 h·ng/ml, with a terminal half-life estimated at 95 h (Table 2). The PK profile of artefenomel in cohort 2 was similar to that in cohort 1. (Both cohorts were administered the same dose of artefenomel.) The mean peak DSM265 plasma concentration in cohort 1 was 5,437 ng/ml, which occurred 2 h postadministration (Fig. 1). The estimated total exposure to DSM265 was 513,573 h·ng/ml, with a terminal half-life of DSM265 estimated at 100 h (Table 2). For cohort 2 (administered half the DSM265 dose of cohort 1), the mean peak DSM265 plasma concentration (3,576 ng/ml) and total exposure to DSM265 (271,276 h·ng/ml) decreased in an approximately dose-proportional manner compared with cohort 1. The elimination half-lives of DSM265 were similar between cohorts. The apparent clearance and volume of distribution associated with the terminal elimination phase were higher for artfenomel compared to DSM265.

**TABLE 2:** Noncompartmental pharmacokinetic analysis

PK parameter^*a*^	Result for:			
	Cohort 1 (*n* = 8)		Cohort 2 (*n* = 5)	
	Artefenomel (200 mg)	DSM265 (100 mg)	Artefenomel (200 mg)	DSM265 (50 mg)
*C*_max_, ng/ml				
Geometric mean	372	5,437	412	3,576
Range	150–597	4,150–6,880	307–522	2,150–4,340
*t*_max_, h				
Median	3	2	5	2
Range	2–5	1–3	1–6	1–2
AUC_0–last_, h·ng/ml				
Geometric mean	3,012	433,771	3,248	231,731
Range	1,110–4,960	328,540–501,880	2,660–5,700	209,470–280,840
AUC_0–∞_, h·ng/ml				
Geometric mean	3,083	513,573	3,317	271,276
Range	1,110–5,090	359,770–633,610	2,680–5,910	239,680–309,310
*t*_1/2_, h				
Geometric mean	95	100	92	97
Range	62–154	76–185	67–137	75–134
CL/*F*, liters/h				
Geometric mean	65	0.19	60	0.18
Range	39–179	0.16–0.28	34–75	0.16–0.21
*V_z_*/*F*, liters				
Geometric mean	8,897	28	7,990	26
Range	4,807–15,963	22–42	4,586–14,271	19–34

a Abbreviations: AUC_0–last_, area under the concentration-time curve up to last time point measure; AUC_0–∞_, area under the concentration-time curve extrapolated to infinity; *t*_1/2_, elimination half-life; *C*_max_, maximum concentration; *t*_max_, time until maximum concentration is reached; CL/*F*, apparent clearance; *V_z_*/*F*, apparent volume of distribution associated with the terminal elimination phase where *F* is bioavailability.

### Pharmacodynamic response: clearance of P. falciparum parasitemia.

**FIG 2:**
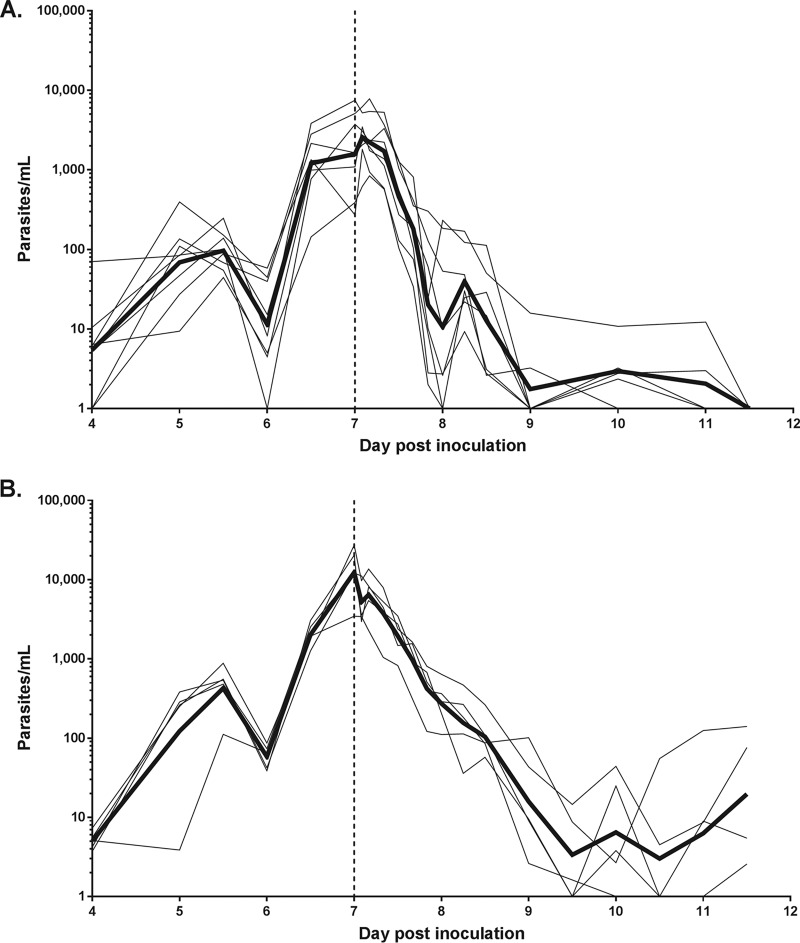
Individual subject parasitemia-time profiles. Subjects were inoculated with ∼1,800 viable parasites on day 0, and a single dose of artefenomel-DSM265 was administered on day 7 (indicated by the vertical dashed line). Cohort 1 received a dose of 200 mg artefenomel plus 100 mg DSM265, and cohort 2 received a dose of 200 mg artefenomel plus 50 mg DSM265. Parasitemia was quantified using qPCR targeting the gene encoding P. falciparum 18S rRNA. Thin lines represent the parasitemia for each subject in cohort 1 (A) and cohort 2 (B), and the bold lines represent the geometric mean. For the purpose of graphing the parasitemia data on a logarithmic scale, the time points at which parasites could not be detected were substituted for with a value of 1 parasite/ml.

Parasites were detected from day 4 postinoculation, with parasitemia reaching the treatment threshold on day 7 (Fig. 2). The geometric mean parasitemias prior to treatment were 1,585 parasites/ml (95% confidence interval [CI], 590 to 4,253) for cohort 1 and 12,339 parasites/ml (95% CI, 4,632 to 32,869) for cohort 2. A rapid reduction in parasitemia was observed in all subjects in both cohorts following artefenomel-DSM265 combination treatment. Recrudescence in asexual parasitemia was observed in 5 of the 8 subjects in cohort 1 between day 19 and day 28 and in all 5 subjects in cohort 2 between day 15 and day 22 (see Fig. S1 in the supplemental material).

The regression models of the log-linear relationship of the parasite decay were significant (*P < *0.001) for 7 of the 8 subjects in cohort 1 and for all subjects in cohort 2. The results from these subjects contributed to the parasite reduction ratio (PRR) calculation. The log_10_ PRRs over 48 h (PRR_48_) were 2.80 (95% CI, 2.56 to 3.04) for cohort 1 and 2.71 (95% CI, 2.57 to 2.85) for cohort 2. The corresponding parasite clearance half-lives were 5.17 h (95% CI, 4.76 to 5.65) for cohort 1 and 5.33 h (95% CI, 5.07 to 5.62) for cohort 2. There was no statistically significant difference in parasite clearance between cohort 1 and cohort 2 when the weighted mean slope of the 2 cohorts was compared using an omnibus test (*P = *0.54).

### Gametocytemia.

**FIG 3:**
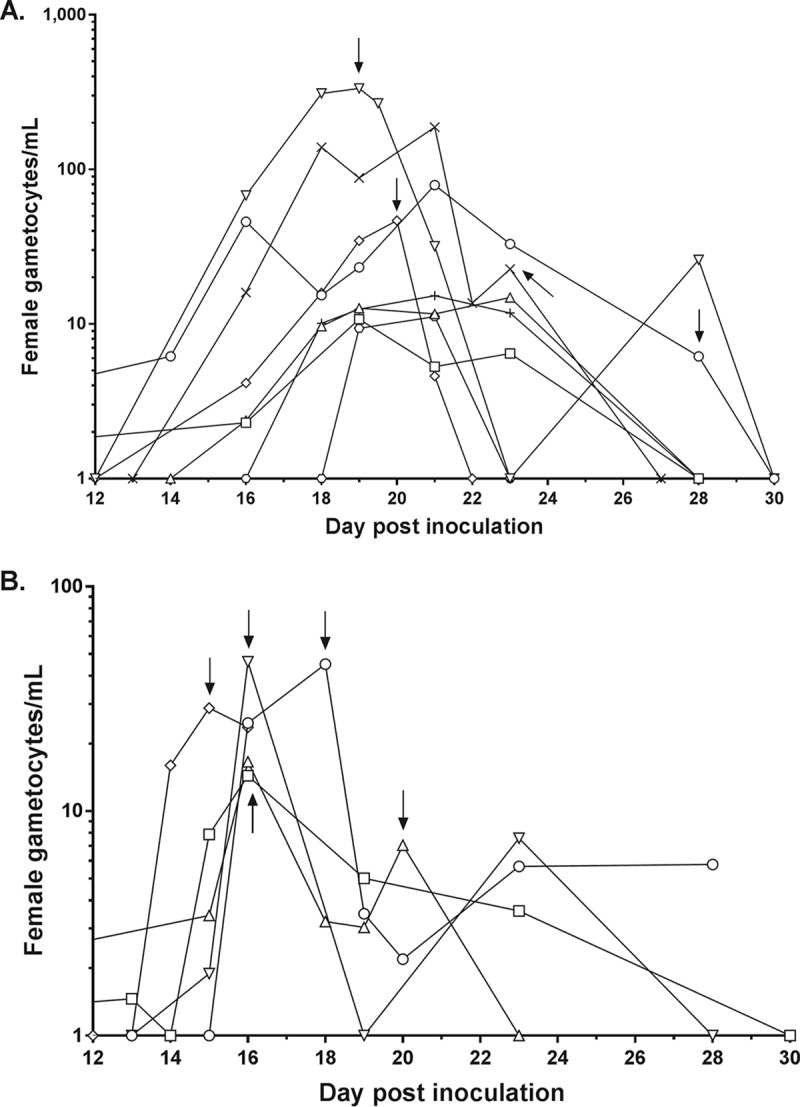
Individual subject gametocytemia-time profiles. Gametocyte density was quantified using qRT-PCR targeting the female gametocyte transcript *pfs25* from 7 days after a single dose of artefenomel-DSM265 was administered. Cohort 1 received a dose of 200 mg artefenomel plus 100 mg DSM265, and cohort 2 received a dose of 200 mg artefenomel plus 50 mg DSM265. Lines represent the gametocytemia for each subject in cohort 1 (A) and cohort 2 (B). Arrows indicate time points at which artemether-lumefantrine treatment was initiated for a particular subject in response to recrudescence of asexual parasitemia. For the purpose of graphing the gametocytemia data on a logarithmic scale, time points at which gametocytes could not be detected were substituted for with a value of 1 gametocyte/ml.

All subjects in both cohorts developed gametocytemia (1 to 330 female gametocytes/ml) after treatment with artefenomel-DSM265 (Fig. 3). Gametocytes were first detected on day 14 postinoculation, 7 days after treatment. Gametocytemia was generally lower in cohort 2 compared with cohort 1; this may have been due to the fact that subjects in cohort 2 received rescue treatment with artemether-lumefantrine earlier than subjects in cohort 1 in response to recrudescence of asexual parasitemia.

## DISCUSSION

This study aimed to investigate the safety, PK, and PD of artefenomel and DSM265 when administered in combination to healthy subjects experimentally infected with blood-stage P. falciparum.

The results show artefenomel-DSM265 combination therapy is safe and well tolerated when administered as a single oral dose up to 200 mg artefenomel plus 100 mg DSM265. No adverse events were considered related to the combination treatment. The good safety profile of this combination therapy is in agreement with the safety findings observed with monotherapy with either artefenomel or DSM265 (2, 4, 9, 10, 14, 18). Additionally, the PK profiles of both artefenomel and DSM265 when administered in combination are similar to those when the compounds are administered separately (2, 4), suggesting that there are no significant PK interactions.

The antimalarial activity of a single oral dose of artefenomel-DSM265 given in combination against low-level P. falciparum parasitemia (289 to 27,312 parasites/ml) was characterized in this study. It is important to note that it was not intended that complete cure would be achieved as PK/PD data from recrudescence are most informative in the pharmacometric modeling exercise that will be reported separately. Initial parasite clearance was rapid in both cohorts, and although clearance occurred at a higher rate in cohort 1 where a higher dose of DSM265 was administered (the log_10_ PRR_48_ were 2.80 for cohort 1 and 2.71 for cohort 2, and the corresponding parasite clearance half-lives were 5.17 h for cohort 1 and 5.33 h for cohort 2), the difference between cohorts did not reach the level of statistical significance. Parasite clearance after artefenomel-DSM265 combination treatment was faster than when each compound was tested individually at similar dose levels in previous IBSM studies: the parasite clearance half-lives were 6.5 h for artefenomel when administered as a single 200-mg dose (2) and 9.4 h for DSM265 when administered as a single 150-mg dose (4). Gametocytemia was observed in all subjects following treatment with artefenomel-DSM265, indicating that combination treatment administered at these doses does not completely inhibit gametocyte maturation.

The optimal characteristics of new combination antimalarial treatments have been defined (19) and guided the selection of artefenomel-DSM265 for this study. In addition to consisting of two drugs with different modes of action to reduce the risk of drug resistance development, an ideal combination treatment will be administered as a single dose in order to avoid issues with compliance that would be associated with a multidose regimen. A single-dose combination treatment will need to clear parasites quickly, but also be effective over a long duration to prevent recrudescent infection. The results presented here suggest that artefenomel-DSM265 combination treatment may meet these requirements. PK/PD modeling of the data obtained in this study to estimate the effective combination dose to be administered in phase 2 field trials will be reported separately.

In conclusion, this study represents the first VIS to investigate a combination of two compounds for the treatment of malaria. We have characterized the safety and antimalarial activity of the new candidate antimalarial compounds artefenomel and DSM265 when administered in combination to healthy subjects experimentally infected with blood-stage P. falciparum. The results support the further clinical development of this combination therapy and will inform the dose selection for future phase 2 field trials. This study also demonstrates that VIS can support the selection of combination antimalarial treatments for late-stage clinical development by providing early data on safety, PK, and PD in a controlled setting.

## MATERIALS AND METHODS

### Study design and participants.

This was a phase 1b, open-label, dose finding study using the IBSM model to characterize the safety, PK and PD associated with coadministration of artefenomel and DSM265. The study was conducted at Q-Pharm Pty Ltd. (Brisbane, Australia) between January and June 2015. Healthy men and women (of non-childbearing potential) 18 to 55 years of age were eligible for inclusion in the study. Individuals were excluded if they had visited an area where malaria is endemic for a period greater than 2 weeks in the past 12 months or had received recent systemic therapy with a drug with potential antimalarial activity. Full inclusion and exclusion criteria are listed in the supplemental material. All participants gave written informed consent before being included in the study. This study was approved by the QIMR Berghofer Medical Research Institute Human Research Ethics Committee (EC00278) and was conducted in accordance with the Declaration of Helsinki. The trial was registered in the ClinicalTrials.gov registry on 20 February 2015 with registration no. NCT02389348.

### Procedures.

The study was planned to be conducted in up to 3 dose cohorts of 8 subjects each. To account for PK differences in the time for each compound to reach peak concentrations, DSM265 was administered 120 min after artefenomel dosing (doses were to be taken within 5 min). The doses that were investigated in cohort 1 were 200 mg of artefenomel and 100 mg of DSM265. These doses were calculated based on previously completed IBSM studies (2, 4) and PK/PD simulation models. The doses used in cohort 2 were 200 mg artefenomel and 50 mg DSM265; these doses were selected following a review of observed safety and parasite clearance kinetics results obtained in cohort 1.

All subjects were inoculated intravenously on day 0 with P. falciparum-infected human erythrocytes (approximately 1,800 viable parasites). Parasite growth was monitored by collecting blood samples and performing quantitative PCR (qPCR) targeting the gene encoding 18S rRNA (20). The threshold for artefenomel-DSM265 combination treatment was ≥1,000 parasites/ml or at the onset of clinical symptoms (the threshold was reached on day 7 in the current study). Subjects received a single oral dose of artefenomel and DSM265, and clearance of parasitemia was measured by qPCR.

Artefenomel mesylate was supplied as 200 mg powder in a bottle by Penn Pharmaceuticals (Gwent, United Kingdom). The powder was suspended in 0.8% polysorbate aqueous solution with Ora-sweet to form a 200-ml suspension for oral administration. The suspension was administered within 40 min of preparation, after the participant had drunk 200 ml of full-cream milk. DSM265 was supplied as 50 mg and 100 mg powder in a bottle by Bend Research (Bend, OR, USA). The powder was suspended in 0.1% methocel A4M, 0.1% polysorbate 80, 0.005% simethicone, 0.05% ethyl vanillin, and 0.5% sucralose to form a 100-ml suspension for oral administration. The bottle was rinsed twice with 70 ml of vehicle following administration of the initial suspension.

All subjects received compulsory rescue treatment with artemether-lumefantrine (Riamet; Novartis Pharmaceuticals, Macquarie Park, Australia) on day 28, or earlier if required. If subjects remained gametocytemic at the end of the study, they were treated with 45 mg primaquine (Primacin; BNM Group, Sydney, Australia).

Safety assessments were performed at screening and at protocol-specified times (see Table S2 in the supplemental material). Safety parameters included AE reporting, physical examination, vital signs, clinical laboratory evaluation, and electrocardiograms.

Blood samples to determine concentrations of DSM265 and artefenomel were taken before artefenomel dosing and at the following time points post-artefenomel dosing: 0.5, 1, 2, 3, 4, 5, 6, 8, 15, 28, 32, 36, 48, 72, 120, 216, 288, 384, 504, 552, and 840 h. Plasma samples were analyzed by liquid chromatography-tandem mass spectrometry (HPLC-MS/MS) as previously described (2, 4).

Parasitemia was measured each morning from day 4 until qPCR results became positive and thereafter at 12-h intervals until treatment initiation. Monitoring then occurred pretreatment and at the following time points posttreatment: 2, 4, 8, 12, 16, 20, 24, 30, 36, 48, 60, 72, 84, 96, 120, and 144 h. Subsequent measurements were approximately three times per week until the end of the study.

Gametocytemia was monitored from 5 days post-artefenomel-DSM265 dosing using quantitative reverse transcriptase PCR (qRT-PCR) targeting *pfs25* mRNA, a transcript preferentially expressed in mature female gametocytes (21). Transcripts per ml of blood were converted to female gametocytes per ml of blood using a standard curve (unpublished data).

### Outcomes.

The primary endpoints defined in the study protocol were the safety and PD associated with coadministration of artefenomel and DSM265. The secondary endpoints defined in the study protocol were the PK of OZ439 and DSM265 and the tendency of treatment to induce gametocytemia.

The PK parameters determined using noncompartmental analysis were the maximum plasma concentration (*C*_max_), the time point when *C*_max_ was reached (*t*_max_), the area under the concentration-time curve up to last time point measure (AUC_0–last_), the area under the concentration-time curve extrapolated to infinity (AUC_0–∞_), the elimination half-life (*t*_1/2_), the apparent clearance (CL/*F*), and the apparent volume of distribution associated with the terminal elimination phase, where *F* is bioavailability (*V_z_*/*F*). The PD variables of interest in this study were the parasite reduction ratio (PRR) and parasite clearance half-life. These provide an estimate of the efficacy of an antimalarial treatment; with the former being the ratio of the parasite density decrease over a 48-h period (expressed as the log_10_ transformed cohort-specific PRR_48_ [log_10_ PRR_48_]).

A PK/PD assessment correlating the kinetics of parasite clearance with the PK profile of artefenomel and DSM265 was also planned in the study protocol. The results of this analysis will be presented in a separate publication.

### Statistical analysis.

The planned sample size of the current study (*n* = 8 per cohort) was comparable with previous P. falciparum IBSM studies and, based on previously published experience, was considered sufficient for obtaining statistically meaningful data on the effects of combined artefenomel/DSM265 on malaria parasite kinetics.

The PD analysis was performed in R version 3.3.0. The PRR and parasite clearance half-life were estimated using the slope of the optimal fit for the log-linear relationship of the parasitemia decay (22). Individual PRR and corresponding 95% CI were calculated using the slope and corresponding standard error (SE) of the optimal regression model. The cohort PRR and parasite clearance half-life were derived using the weighted mean of the optimal slope for subjects with an adequate model fit (*P* < 0.001).

Noncompartmental PK analysis was performed using PKanalix (version 2019R1, Lixoft SAS, Abtony, France). The area under the concentration-time curve was determined using the linear log trapezoidal method in which linear calculation was used before maximum concentration (*C*_max_) and logarithmic formula for after *C*_max_. The slope of the terminal elimination phase (λ*_z_*) was estimated via a linear regression between log concentrations and time. All parameters were summarized using geometric mean or median and range.

## Supplementary Material

Supplemental file 1
