# Supplementary material for: A Single-Dose Combination Study with the Experimental Antimalarials Artefenomel and DSM265 To Determine Safety and Antimalarial Activity against Blood-Stage Plasmodium falciparum in Healthy Volunteers
Source: Antimicrob Agents Chemother. 2019 Dec 20;64(1):e01371-19. doi: 10.1128/AAC.01371-19 (PMC7187626; doi:10.1128/AAC.01371-19)
Supplement: Supplemental file 1 [file AAC.01371-19-s0001.pdf]

SUPPLEMENTARY MATERIAL

A single dose combination study with the experimental antimalarials artefenomel and DSM265 to determine safety and antimalarial activity against blood-stage *Plasmodium falciparum* in healthy volunteers

James S. McCarthy, Thomas Rückle, Suzanne L. Elliott, Emma Ballard, Katharine A. Collins, Louise Marquart, Paul Griffin, Stephan Chalon, Jörg J. Möhrle

Contents

FIGURE S1. INDIVIDUAL SUBJECT PARASITEMIA PROFILES FROM INOCULATION UNTIL END OF STUDY FOR COHORT 1 (A) AND COHORT 2 (B)....2

TABLE S1. INCIDENCE AND SEVERITY OF ADVERSE EVENTS....3

FULL INCLUSION AND EXCLUSION CRITERIA FOR SUBJECT ENROLMENT....4

TABLE S2. SCHEDULE OF EVENTS....9

REFERENCES....11

Figure S1. Individual subject parasitemia profiles from inoculation until end of study for Cohort 1 (A) and Cohort 2 (B)

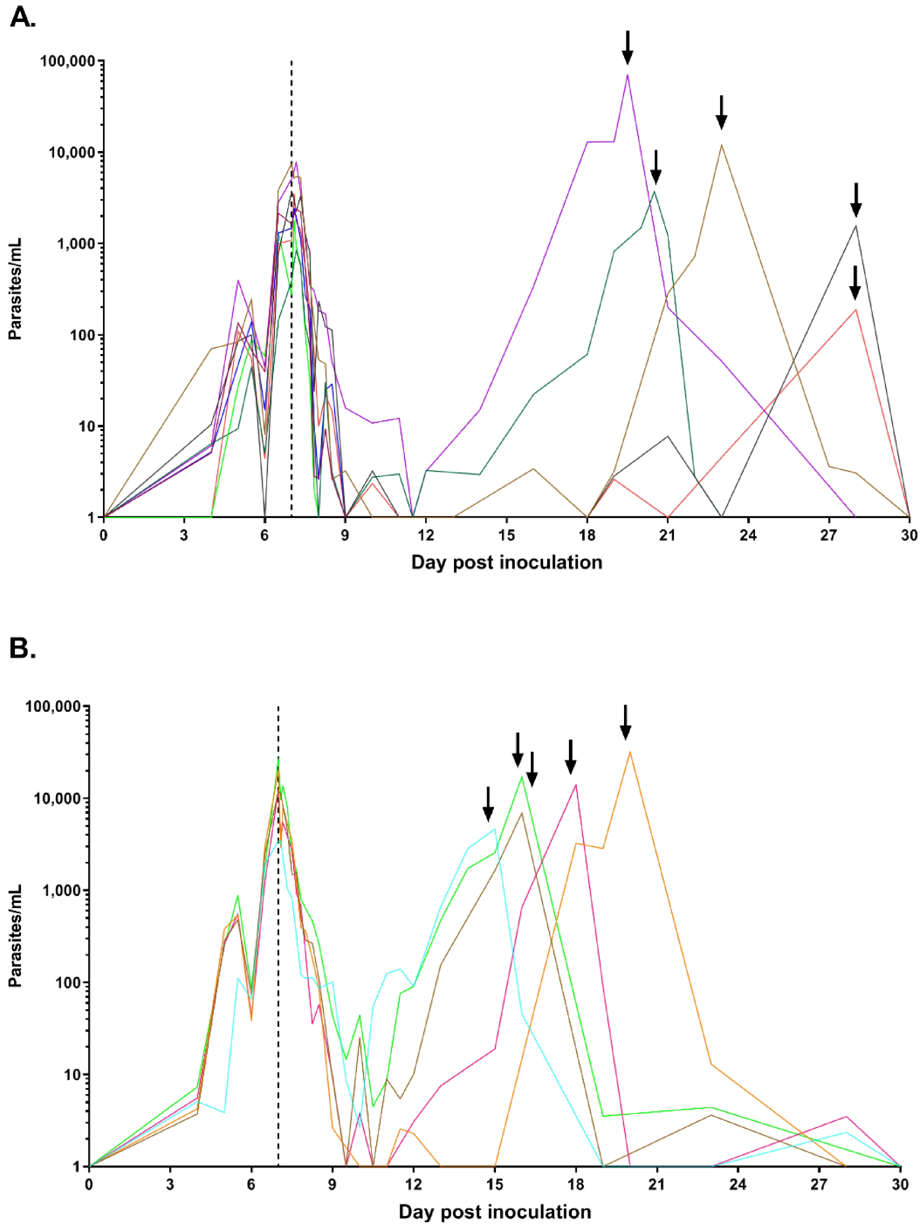

Subjects were inoculated with ~1,800 viable parasites on Day 0 and a single dose of artefenomel/DSM265 was administered on Day 7 (indicated by the vertical dashed line). Cohort 1 received a dose of 200 mg artefenomel/100 mg DSM265, Cohort 2 received a dose of 200 mg artefenomel/50 mg DSM265. Parasitemia was quantified using qPCR targeting the gene encoding *P. falciparum* 18S rRNA. For the purpose of graphing the parasitemia data on a logarithmic scale, timepoints at which parasites could not be detected were substituted with a value of 1 parasite/mL. Arrows indicate the timing of Riamet® administration for each subject in response to recrudescence. Three subjects in Cohort 1 did not recrudesce during the study and were administered Riamet® on Day 28 per protocol (not indicated on graph).

Table S1. Incidence and severity of adverse events

Adverse event	N subjects	N events	Severity (N events)		
			Mild	Moderate	Severe
Abdominal Discomfort *(including Abdominal Cramping, Pain and tenderness)*	2	2	2	0	0
Abnormal urine odour	1	1	1	0	0
Anorexia	3	3	3	0	0
Arthralgia	2	4	3	1	0
Asthma exacerbation*/ Wheezing	2	2	2	0	0
Bruising/ Erythema/ Pain related to cannulation	4	7	7	0	0
Contusion	1	1	1	0	0
Cough	1	1	1	0	0
Dizziness/ Light-headedness	3	3	3	0	0
Erythema	1	1	1	0	0
Fatigue *(including Fatigue and Lethargy)*	9	9	9	0	0
Gastrointestinal Upset *(including vomiting, nausea and intermittent nausea)*	2	2	2	0	0
Headache *(including Headache and Intermittent Headache)*	10	20	16	4	0
Leg tenderness	1	1	1	0	0
Malaise	7	8	8	0	0
Myalgia *(including myalgia in arms, neck, back and shoulders)*	7	10	10	0	0
Tachycardia	3	3	3	0	0
Toothache	1	1	1	0	0
Rhinorrhoea	3	3	3	0	0
Semi-mobile subcutaneous lump	1	1	1	0	0
Skin Lesion	1	1	1	0	0
Sore throat	1	1	1	0	0
Sweats *(including Sweats and Intermittent Sweats)*	4	4	4	0	0
Chills *(including Chills and Intermittent Chills)*	3	4	4	0	0
Fever *(including Fever and Fever Like Symptoms)*	4	6	6	0	0
Elevated Alanine Transaminase	3	3	3	0	0
Elevated Aspartate Transaminase	3	3	2	0	1
Elevated Creatine Kinase	1	1	0	0	1
Elevated Lactate Dehydrogenase	1	1	1	0	0
**TOTAL**		107	100	5	2

*****Asthma exacerbation (mild) was observed in one subject 6 days post combination treatment. This AE was treated with Ventolin and resolved after 5 days. This subject had a medical history of childhood asthma.

Full inclusion and exclusion criteria for subject enrolment

Inclusion Criteria

Demography

1. Adult (male and non-pregnant and non-lactating females) subjects between 18 and 55 years of age, inclusive of those who do not live alone (from Day 0 until at least the end of the antimalarial drug treatment) and be contactable and available for the duration of the trial (maximum of 5 months).

2. Female subjects of childbearing potential must have adequate contraception in place for the duration of the study and extended duration, and have negative results on a serum or urine pregnancy test done before administration of study product. Males prepared to use adequate contraception for the duration of study and extended duration.

3. Body weight, minimum 50.0 kg, body mass index between 18.0 and 32.0 kg/m^2, inclusive.

Health status

4. Certified as healthy by a comprehensive clinical assessment (detailed medical history and complete physical examination).

5. Normal vital signs from supine position to standing:

a. 90 mmHg < systolic blood pressure <140 mmHg,

b. 50 mmHg < diastolic blood pressure <90 mmHg,

c. 40 beats per minute (bpm) < heart rate <100 bpm,

d. Oral body temperature between 35.0 - 37.5°C.

6. Normal standard 12-lead electrocardiogram (ECG) after 5 min resting in supine position taken in triplicate 1 min apart, QTcF≤450 ms with absence of second or third degree atrioventricular block or abnormal T wave morphology.

7. Laboratory parameters within the normal range, unless the Investigator considers an abnormality to be clinically irrelevant for healthy subjects enrolled in this clinical investigation. More specifically for serum creatinine, hepatic transaminase enzymes (aspartate aminotransferase, alanine aminotransferase), and total bilirubin (unless the subject has documented Gilbert syndrome) should not exceed the upper laboratory normal and haemoglobin must be equal or higher than the lower limit of the normal range.

Regulations

8. Having given written informed consent prior to undertaking any study-related procedure.

Exclusion Criteria

Subjects fulfilling any of the following criteria were not eligible for inclusion in this study:

Medical history and clinical status

1. Any history of malaria or participation in a previous malaria challenge study.

2. Must not have travelled to or lived (>2 weeks) in a malaria-endemic country during the 12 months prior to study initiation or planned travel to a malaria-endemic country during the course of the study.

3. Had evidence of increased cardiovascular disease risk (defined as >10%, 5 year risk) as determined by the method of Gaziano et al (1). Risk factors include sex, age, systolic blood pressure (mm/Hg), smoking status, body mass index (BMI, kg/m^2) and reported diabetes status.

4. History of splenectomy.

5. Presence or history of drug hypersensitivity, or allergic disease diagnosed and treated by a physician or history of a severe allergic reaction, anaphylaxis or convulsions following any vaccination or infusion. Lactose intolerant.

6. Presence of or suspected serious chronic diseases at the time of the screening such as cardiac or autoimmune disease (human immunodeficiency virus [HIV] or other immunodeficiency); insulin-dependent Type 1 and Type 2 diabetes and non-insulin dependent diabetes, excluding glucose intolerance if exclusion criteria is met; progressive neurological disease; severe malnutrition; acute or progressive hepatic disease; acute or progressive renal disease; psoriasis; rheumatoid arthritis; uncontrolled asthma; epilepsy or obsessive compulsive disorder; skin carcinoma excluding non-spreadable skin cancers such as basal cell and squamous cell carcinoma.

7. Any surgical or medical condition which might have significantly altered the absorption, distribution, metabolism, or excretion of drugs, or which may jeopardize the subject in case of participation in the study. The Investigator should make this determination in consideration of the subject's medical history and/or clinical or laboratory evidence of any of the following at screening:

- Inflammatory bowel disease, ulcers, gastrointestinal or rectal bleeding 6 months prior to screening;
- Major gastrointestinal tract surgery such as gastrectomy, gastroenterostomy, or bowel resection;
- Pancreatic injury or pancreatitis 6 months prior to screening.

8. Subjects with history of schizophrenia, bi-polar disease, or other severe (disabling) chronic psychiatric diagnosis, including depression or receiving psychiatric drugs or who has been hospitalized within the past 5 years prior to enrolment for psychiatric illness, history of suicide attempt or confinement for danger to self or others.

9. Frequent headaches and/or migraine, recurrent nausea, and/or vomiting (more than twice a month).

10. Presence of acute infectious disease or fever (e.g. sub-lingual temperature ≥38.5°C) within the 5 days prior to inoculation with malaria parasites.

11. Evidence of acute illness within 4 weeks of screening that the Investigator deems may compromise subject safety.

12. Significant inter-current disease of any type, in particular liver, renal, cardiac, pulmonary, neurologic, rheumatologic, or autoimmune disease by history, physical examination, and/or laboratory studies including urinalysis.

13. Subject had a clinically significant disease, or any condition or disease that might have affected drug absorption, distribution or excretion, e.g. gastrectomy, diarrhoea.

14. Participation in any investigational product study within the 12 weeks preceding the study.

15. Participation in any research study involving blood sampling (more than 450 mL/unit of blood), or blood donation to Australian Red Cross (or other) blood bank during the 8 weeks preceding the reference drug dose in the study.

16. Subject unwilling to defer blood donations to the Australian Red Cross Blood Service for 6 months after study completion.

17. Blood donation, any volume, within 1 month before inclusion.

18. Medical requirement for intravenous immunoglobulin or blood transfusions.

19. Subject who had ever received a blood transfusion.

20. Symptomatic postural hypotension at screening, irrespective of the decrease in blood pressure, or asymptomatic postural hypotension defined as a decrease in systolic blood pressure ≥20 mmHg within 2-3 minutes when changing from supine to standing position.

21. History or presence of alcohol abuse (alcohol consumption more than 40 g per day (three standard drinks per day), or drug habituation, or any prior intravenous usage of an illicit substance.

22. Smoking more than 5 cigarettes or equivalent per day and unable to stop smoking during the study.

23. Ingestion of any poppy seeds within the 24 hr prior to the screening blood test (subjects were advised by phone not to consume any poppy seeds in this time period).

24. Excessive consumption of beverages containing xanthine bases, including red bull, chocolate, etc. (e.g. more than 400 mg of caffeine per day or more than 4 cups or glasses per day).

Interfering substance

25. Any medication (including St John's Wort) within 14 days prior to malaria inoculation (Day 0) or within 5 times the elimination half-life (whichever is longer) of the medication. Diazepam was not allowed within 8 weeks prior to inoculation (Day 0).

26. Any vaccination within the last 28 days prior to inclusion.

27. Any corticosteroids, anti-inflammatory drugs, immunomodulators or anticoagulants. Any subject who was receiving or had previously received immunosuppressive therapy, including systemic steroids including adrenocorticotrophic hormone or inhaled steroids in dosages which are associated with hypothalamic-pituitary-adrenal axis suppression, such as 1 mg/kg/day of prednisone or its equivalent or chronic use of inhaled high potency corticosteroids (budesonide 800 µg per day or fluticasone 750 µg).

28. Subject received recent systemic therapy with an antibiotic or drug with potential antimalarial activity (chloroquine, piperaquine, benzodiazepine, flunarizine, fluoxetine, tetracycline, azithromycin, clindamycin, hydroxychloroquine, *etc*.) prior to inclusion.

General conditions

29. Any subject who, in the judgment of the Investigator, was likely to be noncompliant during the study, or unable to cooperate because of a language problem or poor mental development.

30. Any subject in the exclusion period of a previous study according to applicable regulations.

31. Any subject who lived alone (from Day 0 until at least the end of the antimalarial drug treatment).

32. Any subject who could not be contacted in case of emergency for the duration of the trial and up to 2 weeks following End of Study visit.

33. Any subject who was the Investigator or any sub-investigator, research assistant, pharmacist, study coordinator, or other staff thereof, directly involved in conducting the study.

34. Any subject without a good peripheral venous access.

Biological status

35. Positive result on any of the following tests: hepatitis B surface antigen (HBs Ag), antihepatitis B core antibodies (anti-HBc Ab), anti-hepatitis C virus (anti-HCV Ab) antibodies, anti-human immunodeficiency virus 1 and 2 antibodies (anti-HIV1 and anti HIV2 Ab).

36. Subject was found to be glucose-6-phosphate dehydrogenase (G6PD) deficient.

37. Any drug listed in Table 2 of the study protocol (Appendix 16.1.1) in the urine drug screen unless there is an explanation acceptable to the medical investigator (e.g., the subject had stated in advance that he/she consumed a prescription or over the counter (OTC) product which contained the detected drug) and/or the subject had a negative urine drug screen on retest by the pathology laboratory.

38. Positive alcohol urine or breath test.

Specific to the study

39. Cardiac/QT risk:

- Known pre-existing prolongation of the QTcB interval considered clinically significant.
- Family history of sudden death or of congenital prolongation of the QTc interval or known congenital prolongation of the QTc-interval or any clinical condition known to prolong the QTc interval. History of symptomatic cardiac arrhythmias or with clinically relevant bradycardia. Electrolyte disturbances, particularly hypokalaemia, hypocalcaemia or hypomagnesaemia.
- ECG abnormalities in the standard 12-lead ECG (at screening) which in the opinion of the Investigator were clinically relevant or would interfere with the ECG analysis.
- A history of clinically significant ECG abnormalities.

40. Known hypersensitivity to OZ439 or DSM265 or any of its excipients or artemether or other artemisinin derivatives, lumefantrine, or other arylaminoalcohols.

41. Unwillingness to abstain from consumption of citrus (grapefruit, Seville orange, etc.) including their juices as well as quinine containing foods/beverages such as tonic water, lemon bitter and other bitter drinks, from inoculation (Day 0) to EOS.

42. Use of any prescription drugs, herbal supplements, within 4 weeks prior to initial dosing, and/or OTC medication, dietary supplements (vitamins included) within 2 weeks prior to initial dosing (note that diazepam interferes with the analysis of DSM265 and should not have been used for 8 weeks prior to initial dosing). If needed, (i.e., an incidental and limited need) paracetamol is acceptable up to 4 g/day.

On dosing day, and during the blood collection intervals:

- Systemic administration of any drug since the recruitment interview (other than the doses administered in this study) that, in the opinion of the Investigator, could compromise the study.
- Ingestion of any other drug, in the week prior to dosing or during the blood sampling period that, in the opinion of the Investigator, could compromise the study, e.g., through pharmacokinetic or metabolic interactions, or analytical interference. However the Investigator might have permitted the use of paracetamol for the treatment of headache or other pain. If drug therapy other than paracetamol or drug specified in the protocol was required during the study periods, a decision to continue or discontinue the subject's participation was made by the Investigator, based on the nature of the medication and the time the medication was taken.
- Failure to conform to the requirements of the protocol.
- Detection of any drug listed in the protocol in the urine drug screen unless there was an explanation acceptable to the Investigator (e.g., the subject had stated in advance that he/she consumed a prescription or OTC product which contained the detected drug).
- Vital signs outside the reference range and clinically significant.

Subjects were requested to refrain from taking non-approved concomitant medication from recruitment until the conclusion of the study. Any medication taken during the study for treatment of a medical condition or adverse event was to be recorded in the concomitant medication pages in the eCRF.

Subjects who were excluded from participation on study days for any of the above reasons could be eligible to participate on a postponed schedule if the Investigator considered this appropriate.

Table S2. Schedule of Events

**Procedures**	**Screening**	**Challenge Inoculum**	**Malaria Monitoring**			**OZ439/DSM265 Treatment**	**Safety Monitoring**	**Riamet® Treatment**	**Safety Monitoring**	**Final Visit or EOS**
**Day**	**-D28 to –D3**	**0**	**1,2 &3**	**4 AM until PCR**	**Admission**	**Confinement (study day 7-8)**	**Up to 21 days post treatment**	**Timing as outlined in protocol**	**Study day Riamet® (Initial and last day of treatment)**	**Day 42±3**
Informed consent & eligibility	X									
Medical history	X									
Physical examination [a]	X	X		X	X	X	X	X	X	X
ECG [b]	X	X			X	X		X		X
Vital signs [c]	X	X		X	X	X	X	X	X	X
Haematology & biochemistry [d]	X	X			X	X	X	X	X	X
Serology and special tests [e]	X									X
Red cell allo-antibody	X									X
Urinalysis [f]	X									X
Urine drug screen [g]	X	X			X					
Pregnancy (females)	X	X			X					X
**Blood stage challenge**		X								
**Phone call**			X						X	
Unit confinement [h]					X	X				
**Drug treatment**						X		X[k]	X	
**Adverse event**		X	X	X	X	X	X	X	X	X
Malaria qPCR [i]		X		X	X	X	X	X	X	X
OZ439/ DSM265 drug level [j]						X	X	X	X	X
Safety serum storage		X								X

[a] A complete physical examination was performed at screening and the final visit (EOS). An abbreviated physical examination was performed on Day 0, upon admission to the unit and at all morning and evening visits during confinement and where symptoms of malaria were identified.

[b] ECG – 12-lead ECGs were recorded in triplicate at screening, Day 0 pre-inoculum and pre-dose OZ439/DSM265. Single ECGs were to be performed during confinement at 4, 8, 12 and 48 hr post OZ439/DSM265 treatment, prior to Riamet® treatment and at the EOS visit. ECGs could also be taken at safety monitoring visits where clinically indicated.

[c] Temperature (sublingual), respiratory rate, heart rate, blood pressure and mean arterial pressure were obtained at screening, on Day 0 pre-inoculum, 3 times per day during confinement and then as per the schedule for up to 24 days post OZ439/DSM265 treatment, and at the EOS visit. Vital signs could be measured on other visit days where clinically indicated.

[d] See protocol for specific haematology and chemistry tests. Liver function tests were performed 5 and 9 days post-OZ439 and DSM265 where clinically indicated.

[e] Viral and other serology: HIV, Hepatitis B, Hepatitis C, EBV, CMV, G6PD (at screening only).

[f] Urinalysis was performed at screening and at Day 28 or EOS only.

[g] Drug screen: screening, Day 0 pre-inoculum, and on admission to the unit prior to OZ439/DSM265 dosing. Alcohol breath testing was conducted at Day 0 pre-inoculum and on admission to the unit prior to OZ439/DSM265 dosing.

[h] Confinement period 48 hr (planned to be approximately from Day 7 am to Day 9 am).

[i] Malaria qPCR: Day 0 pre-inoculum, then am testing from Day 4 am until qPCR positive for malaria, and then am or am and pm from Day 5 until confinement. During confinement at pre-OZ439/DSM265 dosing, then 2, 4, 8, 12, 16, 20, 24, 30, 36, 48 hr after treatment. Following discharge from the unit, blood samples were taken at 60, 72, 84, 96, 120, and 144 hr after OZ439 dosing. qPCR testing reverted to approximately 3 times per week, until 2 consecutive qPCR tests were negative. For example, blood samples for qPCR could be taken at 216 (D9), 264 (D11) and 312 (D13) hr post OZ439 dosing. qPCR bloods were also taken on the initial and last day of Riamet® dosing, at 384 (D16) and 432 (D18) hr after OZ439 dosing and at the EOS visit. Extra blood was collected for gametocyte specific qRT-PCR from 5 days post-OZ439 and DSM265 dosing.

[j] Blood for pharmacokinetic assessment of OZ439/DSM265 was collected at pre-dose (pre-OZ439 dosing), 0.5, 1, 2 (pre-DSM265 dosing), 3, 4, 5, 6, 8, 15, 28, 32, 36, 48, 72, 120, 216, 288, 384, 504, 552 and 840 hr post OZ439 dosing.

[k] Riamet® was administered to subjects up to 21 days post OZ439/DSM265 if any other criteria in the protocol has not been met. Riamet® doses were taken over 3 days (0, 12, 24, 36, 48, 60 hr).

References

1. Gaziano TA, Young CR, Fitzmaurice G, Atwood S, Gaziano JM. 2008. Laboratory-based versus non-laboratory-based method for assessment of cardiovascular disease risk: the NHANES I Follow-up Study cohort. Lancet 371:923-931.
